# Parenting style and its effect on eating disorders and substance abuse across the young population

**DOI:** 10.1007/s44202-022-00025-7

**Published:** 2022-01-31

**Authors:** Shalina Ramsewak, Numrata Moty, Manish Putteeraj, Jhoti Somanah, Loung-Poorunder Nirmala

**Affiliations:** 1 Psychiatry Department, A.G. Jeetoo Hospital, Port-Louis, Mauritius; 2 Faculty of Law, University of Mauritius, Reduit, Mauritius; 3 School of Health Sciences, University of Technology, Port-Louis, Mauritius

**Keywords:** Parenting style, Eating disorders, Risk factors, Parenting dimensions, Substance abuse, Coping strategy

## Abstract

This article attempts to examine the occurrence of two behavioural changes, namely, substance abuse and eating disorders in order of onset mediated by ineffective parenting styles during young age. The four parenting styles and their consequential behavioural adverse effects are taken as the focal point of this study and are synthesized to provide an outlook on the status of ineffective parenting and deviant offspring behaviours. A review of literature was primarily undertaken to examine the characteristic features of ineffective parenting. A causal relationship was then drawn between the onset of behavioural disorders with an emphasis on substance abuse and eating disorders, along the parenting spectrum. We probed into the order and directionality of the offspring behavioural changes against ineffective parenting. The current available data shows the superimposition of the parenting style spectrum on a bell-shaped distribution of behavioural outcomes as exemplified by authoritarian, permissive and neglectful parenting as a prime determinant of several disorders among the young age; parenting styles being at the extremities of the parenting spectrum. The sweet spot of parenting, mainly associated to the authoritative approach, bears the most positive effect on the growing child. The extreme ends of parenting as per the varying degree of responsiveness and demandingness, generally observed in authoritarian and neglectful parenting precipitate concomitant deviant behaviours cascading from one another; intricately linking substance abuse and eating disorders. A number of studies describe the isolated effects of ineffective parenting on the development of substance abuse and eating disorders during the adolescent period. However, the relationship between both eating disorders and substance abuse is underplayed and need to be stressed upon to tailor behaviour-specific targeted therapies and restore the normalcy of these altered behaviours.

**Supplementary Information:**

The online version contains supplementary material available at 10.1007/s44202-022-00025-7.

## Highlights

- How the parenting style adopted can lead to a concurrent amalgam of disordered eating patterns and substance abuse.
- How disordered eating behaviours and substance abuse are initiated as coping strategies to deal with the consequences of poor parenting.
- Isolation of specific risk factors to deter the development of those deviant behaviours in addition to improvement of parenting methods.

**Supplementary Information:**

The online version contains supplementary material available at 10.1007/s44202-022-00025-7.

## Background

**Fig. 1:**
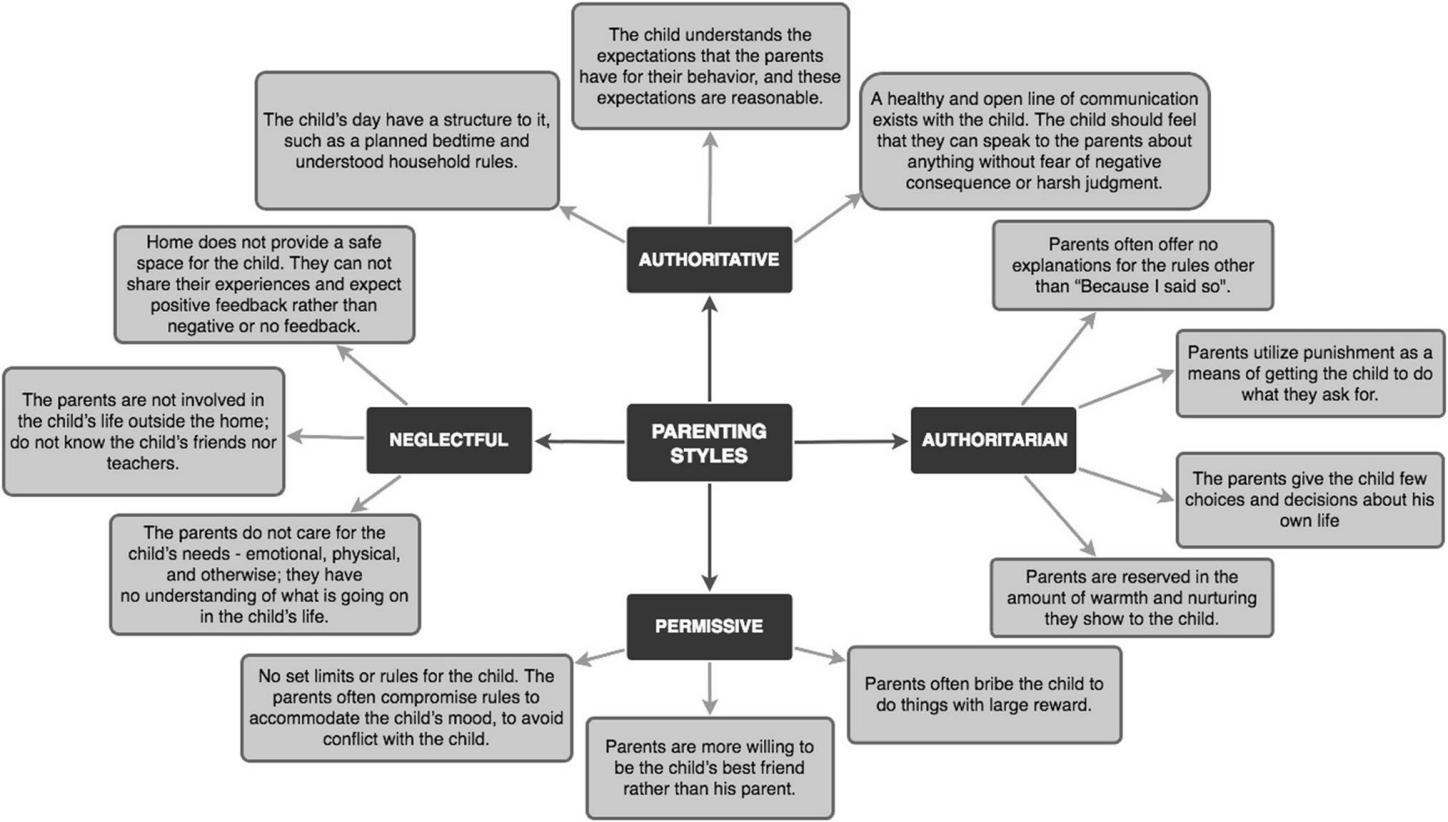
Parenting styles spectrum and their association to child closeness or alienation (*Adapted from Mgbemere and Telles, 2013*)

Understanding parenting and its variants have been critical to enhance the quality and approach taken towards the upbringing of children, with a focus on the values instilled that deter the potential attraction towards social plagues. Although parenting is inexhaustive, the effect of parenting is limited to children aged under 18 years, aligning with the age group for parenting studies [4]; hence, herein after, the young population refers to children prior to 18 years of age. Common parenting styles, namely, authoritarian, authoritative, permissive and uninvolved/neglectful exert their effects based on a continuum of responsiveness and demandingness (Fig. 1) [8, 127]. Authoritarian households consist of authority-driven parents with rigid sets of rules and limited display of affection between parents and child versus warm and responsive authoritative parents with well-defined rules and punishments; permissive parents are indulgent, lenient and affectionate with a lack of direction; while neglectful parents are uninvolved and devoid of direction and warmth [9]. Authoritative versus authoritarian parenting appears to be the most adequate approach as reported by enhanced academic achievement [56], healthy psychological and behavioural functioning [71] as well as good eating habits [117]. Baumrind's model holds many similarities to individual psychological parenting models; imposing authoritative and democratic styles as benefactors over either autocratic or permissive approaches to parenting [36, 94, 107].

## Ineffective parenting and its triggers

Three fundamental dimensions shaping the characteristics of parenting are “warmth versus rejection, structure versus chaos, and autonomy versus coercion” [108, 122]. Warmth determines whether the child feels accepted or rejected by the parents; forging the bond between the child and parents [111]. Structure, in contrast to chaos, represents clear and fair demands while coercion intimidates the child to obey compared to autonomy, the latter reflected by the child’s ability to explore his or her uniqueness [122]. Hence, ineffective parenting predominantly involves a high level of coercion and chaos which is mainly present in dysfunctional households governed by authoritarian and permissive parents [81, 91]. Features of low warmth and variable structure levels are observed in authoritarian and neglectful parents [73]; thereby acknowledging the authoritative style as the most effective parenting method given its protective effect on the child and positive involvement during the child growth mediated by high levels of warmth, structure and psychological autonomy [2, 73, 76, 82]. This is further supported by the detrimental effects of a chaotic household on the socio-emotional adjustments of children [43, 78]. In addition to these three dimensions, researchers also argue that parental incongruency which refers to the opposing parenting style adopted by the individual parents in the same household, can lead to ineffective parenting [45]; and may lead to higher participation in anti-social behaviours in the young aged [131].

Age [79], socio-economic status (SES) [113], mental health [113] and social support [106] of parents are elemental in managing the parenting dimensions and simultaneously affecting the child’s psychosocial development [98]. SES has a multi-factorial effect on the growth of a child, ranging from cognitive stimulation activities and socialisation to parental actions and social interactions [61, 138]. Authoritarian parents from a low SES, holding blue-collar jobs impose strict obedience as a basic requirement [119] in contrast to those originating from high SES households who prone independence [52]. Chronic parental mental health problems consequentially lead to harsh parenting experiences, aberrant interpersonal relationships and increased stress levels which incrementally and cumulatively affect the child [110]. Wang et al. [135] report that poor parental mental health has significant impact on their parenting styles with significant repercussions on their children’s mental health. Depression is closely associated with neglectful parenting [40]. Parents diagnosed with social anxiety disorder tend to be less warm and affectionate towards their offspring [22]. Negative parent–child interaction is also observed in parents suffering from bipolar disorder [32, 57, 120].

The intricate relationship between SES, mental health and parenting style, as investigated by Topham et al. [129], predicts an increased likelihood of irregular offspring behaviour induced by permissive depressed mothers versus non-depressed authoritarian ones. This concurs to a certain extent with the ‘maternal maturity hypothesis’ stating that younger mothers are less likely to provide their children with skilled and appropriate parenting, leading back to SES and parenting style [17, 70].

## Consequential effects of ineffective parenting

A good family environment is central to the proper development of children, with parents having the paramount responsibility to discipline the child to mediate a successful transition into adulthood [37]. Permissive parenting induces a higher probability of low self-esteem, educational difficulties, school delinquency, and substance use in children [109]. Conversely, strong parental support and autonomy as depicted by authoritative parenting leads to higher self-esteem and life satisfaction, lower depression [28, 44, 88, 93, 100], and positively impacts adolescents’ educational achievement [56]. Permissive parenting potentially increases the likelihood of engaging in impulsive behaviours such as alcohol abuse [6]; risky sexual behaviour especially among females [38]; and heightened display of disruptive behaviours by males [105] due to the lack of parental behaviour control [121]. Children living with authoritarian parents are more likely to encounter interpersonal problems, resulting from a declining self-confidence and an increased prevalence of depressive states [88, 114]. A meta-analysis conducted by Hoeve et al. [51] validated the onset of criminal behaviour among the changes reflected in children raised by neglectful parents, further substantiated by social disorganization and poor parental-offspring social transactions. This adds to traits of emotional withdrawal, fearfulness, anxiety, and poor academic performance, with increased risk of substance abuse in such families [33] due to the lack of family cohesion [96].

### Parenting styles and eating disorders

Eating disorders are characterized by chronic disturbance in the eating patterns of an individual along with significant distress in the important areas of functioning [39]. Disordered eating habits include frequent unhealthy dieting, laxative use, binge-eating, caloric restriction, anorexic and bulimic behaviours [1]. Past studies have successfully demonstrated the predominant onset of disordered eating habits at a young age [74] which tends to persist into adulthood [99]. Obesity among children is considered to be an ‘epidemic’ given its sharp inclined prevalence. Disordered eating habits are gender-specific, affecting more females than males [68, 126]. Biologically, women have a lower rate of basal fat oxidation which promotes more fat storage; contributing to disordered eating behaviours [16]. Objectification of the female gender is thought to be a mediating factor of eating disorders as reflected by a rise in thin waistline adoption [68]. This phenomenon prevails as a result of the sexualisation of women’s bodies and the evaluation of their self-worth based on their appearance and/or bodies when contrasted against body ideals portrayed across social media [3]. Hence, women resort to different strategies including disordered eating behaviours to achieve the ideal body [132].

Isolation of a single factor as the trigger for disordered eating behaviour is not permissible. Internal triggers such as genetic predisposition, neurobiological mechanisms inclusive of cognitive and behavioural attributes can be potential risk factors [83, 102]. External factors modulating the prevalence of disordered eating habits refer to sociocultural traits such as societal and mass media pressure particularly through Social Networking Sites (SNS) [77], which more than often tips the cultural standards towards ‘size zero’ features [68].

Importantly, parental psychological conditioning is not limited to the cognitive and behavioural characteristics of a child but also co-extends to the physiological and metabolic features [14, 115]. In recent years, there has been a growing interest probing into the association between parenting style and eating disorder (Table 1). The development of an irregular eating pattern can be induced by parenting [14, 25]. The authoritarian and permissive styles are more significantly linked with eating disorders in contrast to authoritative parenting [49]. Indeed, a recent review of literature by Kiefner-Burmeister and Hinman [64] has demarcated authoritative parenting as being protective of obesity in children and supportive of healthy eating habits. Children are more likely to develop disordered eating habits if the fatherly figure is perceived as authoritarian over authoritative [41]. According to Momeni and Amiri [92], there is a significant negative relationship between the authoritative style of parents and anorexia nervosa (AN) among female adolescents [18]; hence endorsing the relationship between parental warmth and good eating behaviour. Alternately, incongruent parenting patterns have been linked to obesity in adolescents as reflected by a deregulation of the eating patterns related to contradictory responses to eating cues from the parents [13]. Incongruency can also trigger disordered eating behaviour, with adolescents indulging unhealthy snacking, if the mother and the father adopt contrasting parenting styles [45]. Being on the low end of parental control, permissive parenting style is often considered as being the over-indulgent parenting style, leading to a higher risk of eating disorders [49, 116]. Binge-eating practice in females is more prominent when authoritarian (17.1%) and permissive (10.7%) parenting styles are adopted versus authoritative parenting (8.3%) [139]. This synthesis of literature on eating disorders and parenting anchors authoritative parenting as a mitigator versus the remainder ineffective parenting styles as potentiators.

**Table 1:** Connecting parenting styles and eating disorders

Authors	Findings
Klesges et al. [66]	↑ Effects of parental monitoring (threats and actual) on 53 children’s obesity status, ↓ calorie intake and non-nutritious food
Wake et al. [134]	↑ Paternal control ↓ BMI while, ↑ Parental neglect and permissiveness leads to ↑ BMI
Olvera and Power [104]	Mexican American children: ↑ Maternal indulgence, ↑ risk of obesity
Sleddens et al. [123]	Children from authoritative parenting had ↑ healthy eating patterns and ↓ BMI, compared to children from neglectful, permissive and authoritarian parenting
Johnson et al. [59]	Parents of Anorexics; Mother were ↑ critical and dominating (authoritarian) and father were ↑ permissive
Ingoldsby et al. [58]	Neglectful parenting, ↑ chances of developing bulimia
Depestele et al. [35]	Household with ↑ maternal control (authoritarian), ↑ binge-eating purging behaviours

### Substance abuse in the Young population

Substance abuse has reached a critical status across the global population transcending the transboundary barriers. There has been a growing concern with regards to adolescents inclined towards substance abuse as early as 13 years of age [62, 136]. The psychoactive substances consumed vary from the ten classes of drugs and other unknown substances which altogether have the ability to modulate the reward system; tempering with the normal neurobiological development of the mesolimbic and mesocortical circuitry to alter behaviour [128].

Substance abuse develops over a spatio-temporal scale and is mediated via social transmission; the latter referring to the wilful participation of the host in the process. For instance, there is an increased risk of substance abuse initiation if one spouse in a family household is registered as a user [63]. The temporal nature of substance abuse is best described using the ‘Gateway Drug Theory’, which defines the addiction process as an escalation from alcohol, tobacco or cannabis to more addictive psychoactive substances during later life stages [101]. This theory applies primarily to the adolescent years as reported by Kirby and Barry [65] with 12th grade students shifting towards illicit drugs during adulthood after being exposed to tobacco and cannabis. Gender-based differences in substance abuse are also highly prevalent although the gender gap appears to narrow down when comparing adolescent girls and boys [84]. Men are twice at risk to indulge in substance abuse as compared to women, the latter who progress faster from initial exposure to substance use disorders through telescoping [29, 69, 72].

The complexity of substance abuse as a mental disorder is attributed to the interplay of various genetic and environmental risk factors [60]; however, the prevention of the disorder at a young age is plausible by cushioning the modifiable risk factors [48]. Imitation and social learning as part of the environmental risk factors can promote substance use disorders [124]. Lack of communication and support [26], parental drug abuse [53], social disorganisation [19], academic failure [31] and rejection by peers [87] are the most common determinants observed in substance abusers. Family functioning, through the involvement and emotional expressiveness of the parents, has proved to be elemental in attenuating the risk of substance abuse in the offspring [26]. Low SES households where parental guidance is poorly observed throughout adolescence increase the affinity towards negative peers [24]; statement in line with Mendez et al. [87] showing that adolescents become victims of drug abuse as a means to peer group submission or acceptance. The contrary, i.e., peer rejection leading to increased psychological stress and consequential substance abuse among adolescents also stands true [31]. The inter-relationship among these factors termed as gene-environment correlation [67] may precipitate substance use disorders.

### Parenting styles and substance abuse

Adolescence is a critical period of life during which, vulnerability to substance use disorders may be stimulated [133]. Parenting styles influence children in their decision-making process across adolescence; hence, control to a certain extent, the exposure to illicit substances [12]. If protective factors boosting self-efficacy and self-esteem among other characteristics within the parenting style adopted are present, the onset of substance use disorder can be mitigated; while the opposite also remains for ineffective parenting [137].

Authoritative parenting is considered to be the ideal method to nurture maturity and generate the best results in terms of the lowest substance abuse rates [10, 12]. It relies on consistent discipline, warmth and sensitivity towards their children, which exerts a positive force on the growing children and culminates in both parent and child communicating on the same wavelength [20]. Children victims of harsh punishment by authoritarian parents have an increased risk of using substances as a coping mechanism [12]. Permissive parenting serves as a good predictor of adolescent substance abuse given the indulgent nature of the parents and the lack of behavioural demands from the child leading to poor outcomes with respect to substance abuse [21, 118]. Neglectful parents fail to set limits and do not provide their children with adequate warmth, rendering them more vulnerable to substance abuse [10]. A Brazilian prospective study involving 99 adolescents reported 30% and 28% respondents exposed to authoritative and neglectful parenting respectively; with adolescents from the latter having more trouble abstaining from substance abuse [11]. These findings align with reports from Martínez-Loredo et al. [80] demonstrating a strong link between neglectful parenting and adolescent alcohol abuse with an odds ratio (OR): 2.14; 95% confidence interval (CI): 1.18–3.86 (p = 0.012). The ascending order of substance abuse predictability with respect to parenting style can be inferred to be authoritative, authoritarian, permissive and neglectful, the latter being the most potent precipitator of substance abuse among the young population [97]. Through the extensive research undertaken linking parenting and substance abuse, the modifiable risk factors leading to this deviant behaviour is almost entirely englobed by the parenting style adopted within the household and plays a prominent role in guiding the trajectory of the child’s development.

## Connecting the dots between ineffective parenting styles, substance abuse and eating disorders

Eating disorders and substance abuse is exclusive in some adolescents but can exist simultaneously as supported by the strong correlation between eating disorders and substance abuse [15, 46]. Approximately 50% of individuals with eating disorders also abuse alcohol or illicit drugs [112]. Similar findings have been documented by Denoth et al. [34] in a cross-sectional study involving 33,185 Italians aged between 15 and 19 years old; with a higher proportion of abnormal-weight respondents (20–40%) abusing illicit substances over the normal population. Both eating disorders and substance abuse are used to numb away emotional distress, anxiety, depression, apprehension and psychological trauma; and serves as a coping mechanism for pain control [5, 55]. Children exposed to acute or chronic stressors related to weight gain resort to substance abuse as a remedial action to suppress appetite [54]. Alternately, studies have shown that binge eating is often accompanied by heavy alcohol consumption as a coping strategy during unpleasant situational events [5]. Interestingly, the risk of developing substance dependence or abuse is 3.4 times higher in subjects engaged in recurrent binge eating versus normal participants [89]. Women diagnosed with AN during their adolescence are more prone to develop alcohol-related problems which appear to persist over adulthood even if the eating disorder is treated [95]. Overlooking the myriad of common environmental and genetic factors between both behavioural disorders, parenting style appears to be a key determinant and trigger to substance abuse and eating disorders [83].

A longitudinal study conducted by Chen et al. [27] from 1989 to 2004 involving 16,882 participants provided critical data related to positive parenting and its inhibitory effects on eating disorders and substance use. The findings revealed a positive association between the authoritative approach characterized by a consolidated parent–child relationship and offspring satisfaction, and a lower risk of eating disorders and marijuana use, while promoting good physical and mental well-being. The data concurred with Enten and Golan [41] stating that dietary habits and body image are affected by parenting, familial milieu, parent–child bonding and communication. Haines et al. [47] demonstrated a low association (AOR = 0.73, CI = 0.60–0.88) between highly functioning families and obesity among the children, and Hock et al. [50] posited the father–youth bonding to be of utmost importance in shielding against substance abuse.

Parenting styles are also intricately linked to early maladaptive schemas (EMS) which can consequentially lead to eating disorders and substance abuse. Permissive parenting is positively associated with the development of EMS which may promote psychosocial outcomes inclusive of eating disorders [23] and addiction [7]. Contrastingly, the authoritative style negatively correlates to the generation of EMS given the relative absence of household characteristics of rejection, abuse, and instability among others [42]. Although beyond the purview of this review, the EMS dimension related to the other characteristics of parenting and its impacts on psychosocial behaviours could be further explored as supporting elements to the concept of ‘wrong’ parenting and adverse child development.

## Conclusion

This review critically portrays the different dimensions of parenting and focuses on the styles which precipitate aberrant behaviours inclusive of eating disorders and substance abuse. Parenting forms the basis of the psychological development of a child. It can be considered as a yardstick to shape and prepare the offspring to face the future challenges in life by developing good self-esteem and coping strategies which are primordial in problem-solving and decision making in later life for the betterment and well-being of the child [86]. Parental warmth and control are of utmost importance in preventing substance abuse in children [50]. There has been strong evidence showing a link between ‘bad’ parenting practices during the early childhood developmental stages to the onset of eating disorders and substance abuse [75]. However, parenting being multi-dimensional, characteristics such as parenting practices, discipline strategies and parental psychopathology involved in the child development cannot be disregarded [85]; along with its consequential effects on the child habits including substance abuse and eating disorders.

## Research gap and future direction

**Fig. 2:**
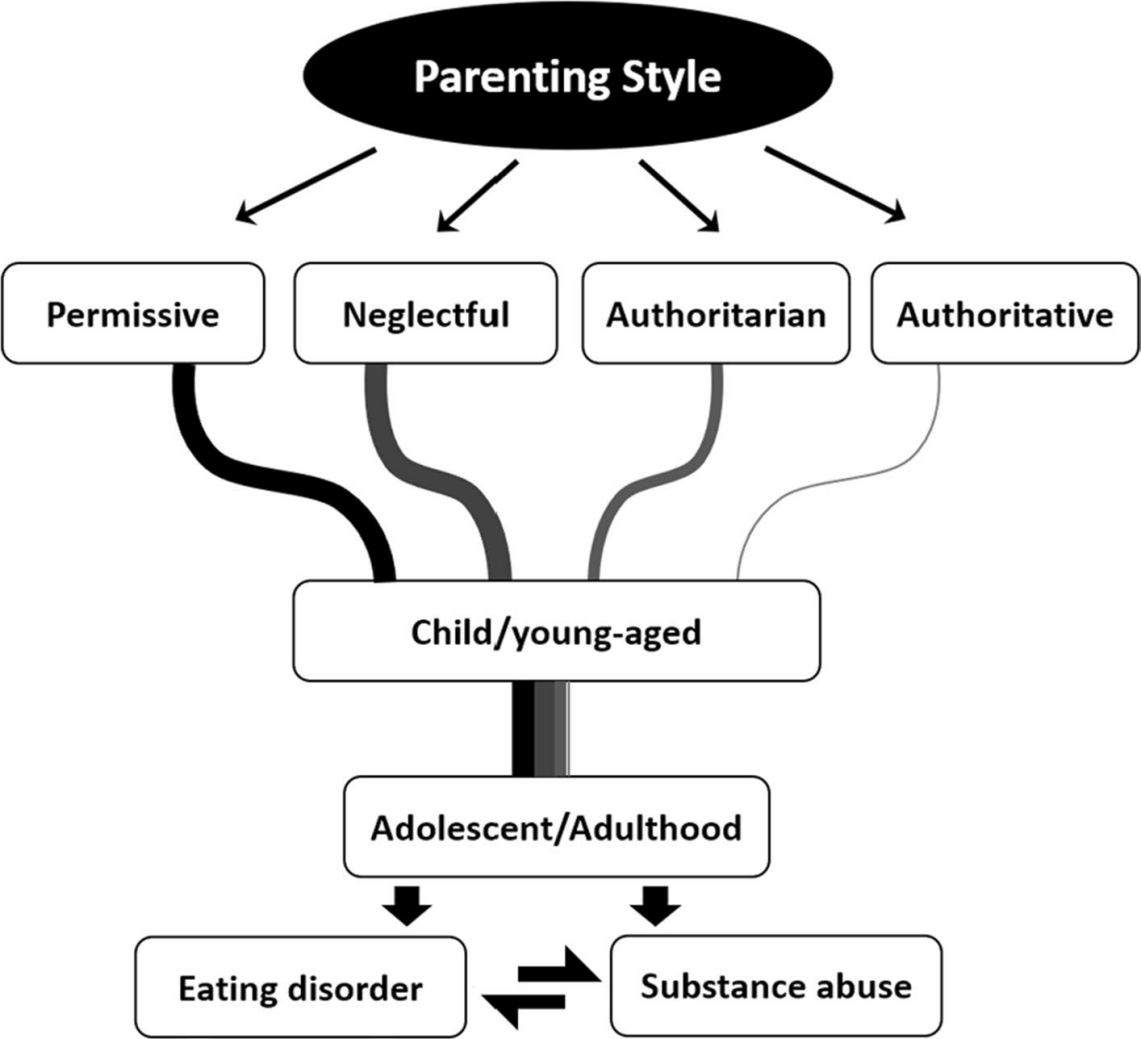
Exposure of the young-aged to differential parenting styles and behavioural consequences during adolescence/adulthood stages

The present study tackles the influence of the different parenting styles on young individuals’ proneness to develop eating disorders or substance use disorders. Although at this stage, ineffective parenting and the ranked occurrence of the mentioned behavioural disorders, i.e., order of onset between eating disorders and substance abuse cannot be determined, it can be inferred that the cascading effect of one condition may lead to the other in adolescents. Data highlighting those two simultaneous deviant behaviours remains scarce to this date and requires more attention across the scientific community to precipitate the demand for parenting training programs (PTPs) which is limited in the societal sphere. PTPs can be multi-dimensional with the inclusion of early signs detection towards deviant behaviour, regulation of parental behaviour and characteristics, as well as behavioural family interventions to enhance communication strategies and effects, while creating more conducive environments within households to nurture healthy development. The level of influence of individual parenting styles, i.e., the tolerable threshold to trigger those behavioural disorders still remains unclear given the potential hybridization across the variants; but permissive and neglectful parenting appears to be much more influential in both cases (Fig. 2). Importantly, this review focused on conventional households and has not taken into consideration the aspect of inclusivity whereby same-sex parents, co-parenting and re-composed families might act as potent co-factors and reflect different degrees while alternating parenting style adoption.

In recent years, helicopter parenting, coined by Cline and Fay [30] seemed to have gained some ground. This style of parenting, consisting of “hyper-involvement” in children’s activities, has been associated with poor social skills and self-efficacy [125]. This particular finding is interesting, for recent studies show that indulgent/permissive parenting seems to result in more positive outcomes in youngsters with higher self-esteem and good educational performance [11], shedding more light on the degree of responsiveness required and also the developmental timeframe for such variation. The social fabric has witnessed changes of the highest magnitude with the drastic measures imposed by multiple countries since the coronavirus (Covid-19) outbreak in 2020 which may seem to favor permissive parenting style as reported by the positive effects on the behavioural traits in Japanese children [90]. However, the conventional authoritarian style remains connected to negative outcomes such as the child’s mental health, screen time and sleep quality among others [103]. ‘Forced’ parental involvement during the pandemic over the extended durations of quarantine and confinement has been an ‘eye opener’ for many, mostly in determining the efficiency of their parenting styles and effect on their children’s performance and behaviour [130]. The impact of such Covid-19 social measures warrants further research on multiple fronts when it comes to the young population and behaviours inclusive of eating habits and substance use as well as the hybridization of parenting styles and potential observed changes in parenting as a result of the ‘new normal’.

## Supplementary Information

Below is the link to the electronic supplementary material.

Supplementary file1 (DOCX 30 kb)

## Data Availability

Data sharing is not applicable to this article as no datasets were generated or analysed during the current study.

## Abbreviations

AN: Anorexia nervosa
EMS: Early maladaptive schemas
OR: Odds ratio
SES: Socio-economic status
SNS: Social networking site
