# Supplementary material for: Parenting style and its effect on eating disorders and substance abuse across the young population
Source: Discov Psychol. 2022 Jan 31;2(1):9. doi: 10.1007/s44202-022-00025-7 (PMC8802280; doi:10.1007/s44202-022-00025-7)
Supplement: Supplementary file 1 — Supplementary file1 (DOCX 30 kb) [file 44202_2022_25_MOESM1_ESM.docx]

**Additional: Methodology**

A review of literature was primarily undertaken to examine the characteristic features of ineffective parenting. Concepts of a systematic review were used with the exclusion of the blind selection process given the narrative nature of the article. The keywords used for literature search were as follows: parenting style, eating disorders, exercise, substance abuse, young population, children, risk factors were used as strings using Boolean and proximity operators through three major databases namely, Web of Science, PsyInfo and Pubmed Central. Given the broad nature of parenting as reported by 7430 articles on PsyInfo, the data was crossed with stringed search terms on the other databases to narrow down the reference articles based on the core concepts of the study. The inclusion criteria as a representative of the population were mainly children and adolescents which may have developed the outcome, i.e., eating disorders and/or substance abuse in that developmental stage or later on in adulthood. The intervention under investigation was parenting style. Out of the 216 articles primarily selected, filters applied referred to duplication of articles, selection based on inclusion criteria and the prioritized outcome as specified using the PICO approach, leading to a final selection of 114 articles. Two authors were involved in the primary selection of the articles, while the other 2 authors were provided with the same set for validation; out of which 136 and 118 articles were filtered. Validation of the selected articles was based on the relevance to the topic of parenting style and the outcomes of interest. The articles which were not commonly selected was reviewed by the 5^th^ author and agreement was reached on the decision to retain only the common filtered articles.


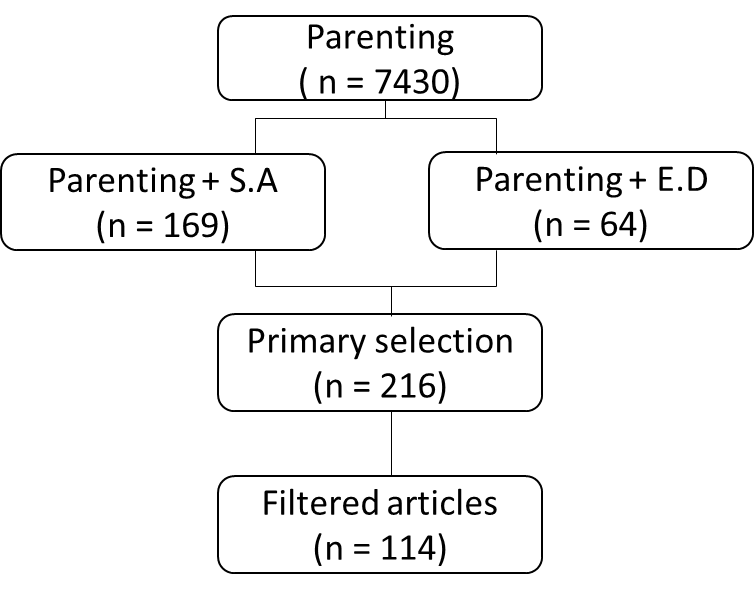


**Figure 3**. Article sourcing and filtering process. A primary search on databases was further streamlined through the different application of filters generating a final 114 articles. (S.A: *substance abuse*; E.D: *eating disorder*)
